# Construction of HBV gene-related prognostic and diagnostic models for hepatocellular carcinoma

**DOI:** 10.3389/fgene.2022.1065644

**Published:** 2023-01-04

**Authors:** Keqiang Ma, Hongsheng Wu, Lei Ji

**Affiliations:** ^1^ Department of Hepatobiliary Pancreatic Surgery, Affiliated Huadu Hospital, Southern Medical University (People’s Hospital of Huadu District), Guangzhou, China; ^2^ Department of Hepatobiliary Pancreatic Surgery, Renmin Hospital Hubei University of Medicine, Shiyan, China

**Keywords:** LIHC, HBV, molecular subtypes, prognostic characteristics, immune microenvironment

## Abstract

**Background:** Hepatocellular carcinoma (HCC) is a main cause of malignancy-related death all over the world with a poor prognosis. The current research is focused on developing novel prognostic and diagnostic models of Hepatocellular carcinoma from the perspective of hepatitis B virus (HBV)-related genes, and predicting its prognostic characteristics and potential reliable biomarkers for Hepatocellular carcinoma diagnosis.

**Methods:** As per the information related to Hepatocellular carcinoma expression profile and the clinical data in multiple public databases, we utilized limma for assessing the differentially expressed genes (DEGs) in HBV vs non- hepatitis B virus groups, and the gene set was enriched, analyzed and annotated by WebGestaltR package. Then, STRING was employed to investigate the protein interactions. A risk model for evaluating Hepatocellular carcinoma prognosis was built with Lasso Cox regression analysis. The effect patients receiving immunotherapy was predicted using Tumor Immune Dysfunction and Exclusion (TIDE). Additionally, pRRophetic was used to investigate the drug sensitivity. Lastly, the Support Vector Machine (SVM) approach was utilized for building the diagnostic model.

**Results:** The Hepatocellular Carcinoma Molecular Atlas 18 (HCCDB18) data set was utilized for the identification of 1344 HBV-related differentially expressed genes, mainly associated with cell division activities. Five functional modules were established and then we built a prognostic model in accordance with the protein-protein interaction (PPI) network. Five HBV-related genes affecting prognosis were identified for constructing a prognostic model. Then, the samples were assigned into RS-high and -low groups as per their relevant prognostic risk score (RS). High-risk group showed worse prognosis, higher mutation rate of TP53, lower sensitivity to immunotherapy but higher response to chemotherapeutic drugs than low-risk group. Finally, the hepatitis B virus diagnostic model of Hepatocellular carcinoma was established.

**Conclusion:** In conclusion, the prognostic and diagnostic models of hepatitis B virus gene-related Hepatocellular carcinoma were constructed. ABCB6, IPO7, TIMM9, FZD7, and ACAT1, the five HBV-related genes that affect the prognosis, can work as reliable biomarkers for the diagnosis of Hepatocellular carcinoma, giving a new insight for improving the prognosis, diagnosis, and treatment outcomes of HBV-type Hepatocellular carcinoma.

## Introduction

Incidence of hepatocellular carcinoma (HCC) is the sixth highest among some other frequently known cancers, with mortality ranking the fourth highest. HCC makes up 80–90% of the global primary liver cancer (Bray et al., 2018). The majority of patients diagnosed with HCC have local progression or distant metastasis because of a lack of identifiable symptoms at the early stages (Ranganathan et al., 2020). Despite significant advancements in medical and surgical procedures (Forner et al., 2018), 5-year survival rate of HCC is only about 18% (Villanueva, 2019), with a poor prognosis (Hartke et al., 2017; Jiang et al., 2017; Nishida and Kudo, 2017). Hence, finding new therapeutic targets for HCC treatment has crucial significance. Moreover, when evaluating the survival prognosis of individuals, it is also necessary to coordinate the clinical-pathological characteristics of the genome.

Primary risk factors of inducing HCC are Hepatitis B (HBV) and C (HCV), followed by exposure to aflatoxin B1, alcohol, and obesity (de Martel et al., 2015; Bray et al., 2018). Integration of HBV DNA into hepatocytes for persistent viral infection, which could result in chronic hepatitis B infection, ultimately causing HCC. Evidence has shown that HBV proteins directly affect multiple cellular biological processes, and that some of the proteins could stimulate malignant transformation of hepatocytes (Ayub et al., 2013). Chronic hepatitis B-related HCC makes up for more than 80% of all HCC cases (Villanueva, 2019). Despite the progress made in the early diagnosis, prevention, and standard treatment interventions (such as surgery, radiotherapy, chemotherapy, or tailored treatment strategies) in the past decade, total 5-year overall survival of HCC is unfavorable, which may be resulted from its aggressive behaviors as well as the histopathological and molecular heterogeneity of molecular characterization and targeted treatment strategies. Moreover, most HCC patients are diagnosed at a more advanced stage of the disease, which often leads to a worse prognosis. Therefore, to better construct a prognosis and diagnostic model of HBV-induced HCC still has crucial significance for a timely diagnosis of HCC.

In the current research, data of patients with HBV-related HCC from the Cancer Genome Atlas (TCGA), HCCDB18, and GSE14520 were collected for the identification of DEGs between non-HBV-infected and HBV-infected people. We highlighted the biological role and identified interacted modules of differentially expressed genes (DEGs). Finally, a prognostic and diagnostic model was developed using the HBV gene in HCC and validated for its reliability and effectiveness. In conclusion, this report offered a potential indicator for assessing the molecular mechanism of HCC progression and development and helped study the effect of immunotherapy in detail, providing new insight for timely diagnosis, prognosis prediction, and immunotherapy of HCC.

## Methods

### Data collection and pretreatment

Sangerbox platform (http://vip.sangerbox.com/) supported the current research analyses (Shen et al., 2022). We took the latest clinical follow-up, expression data, and mutation data of HCC patients’ tissues from the TCGA (http://cancergenome.nih.gov/abouttcga) in 30 April 2022 (Tomczak et al., 2015). After excluding the samples having no data on clinical follow-up, survival time, and status, the RNA-sequencing (RNA-Seq) data contained 365 samples after preliminary identification. Next, the data Ensembl ID was transformed into gene symbol, with the median expression value of a gene corresponding to numerous gene symbols being taken.

The TCGA mutect2 software’s mutation data set were obtained. 2564 genes, those with a mutation frequency of more than three were chosen. The genes with substantial high-frequency mutations were chosen using the Fisher test from each subtype, and the threshold for selection was *p* < 0.05.

HCCDB18 data was collected online from the website (http://lifeome.net/database/hccdb/home.html) on 30 April 2022. Similarly, we eliminated the samples without data on expression profile, survival time, status, clinical follow-up. After identification, 203 samples in total were selected for this study.

Gene Expression Omnibus (GEO) database (https://www.ncbi.nlm.nih.gov/geo/) gave us the GSE14520 data, and the HCC patients’ chip data set with survival time was chosen. The download time was 30 April 2022. The data set excluded the samples having no data on clinical follow-up, survival time, and status. After identification, it finally contained 221 samples. Then, the median expression of multiple Gene symbols was considered after transforming the Ensembl in the data into a Gene symbol. See Table1 for the clinical statistical information sample after the pretreatment of the three groups of data.

**TABLE 1:** The clinical information of three datasets.

Clinical features	TCGA-LIHC	HCCDB18	GSE14520
OS			
0	235	168	136
1	130	35	85
T Stage			
T1	180	33	
T2	91	96	
T3	78	59	
T4	13	15	
TX	3		
N Stage			
N0	248		
N1	4		
NX	113		
M Stage			
M0	263		
M1	3		
MX	99		
Stage			
Ⅰ	170		93
Ⅱ	84		77
III	83		49
Ⅳ	4		
X	24		2
Grade			
G1	55		
G2	175		
G3	118		
G4	12		
GX	5		
Gender			
Male	246	153	191
Female	119	50	30
Age			
≤60	173	43	181
>60	192	160	40
HBV			
YES	22	53	
NO	159	29	
Unknown	184	121	

### DEGs and functional enrichment analysis

The DEGs were analyzed using limma (Ritchie et al., 2015) and filtered using the criteria of |Fold Change (FC)| > 1.2 and *p* < 0.05. Using the ‘WEB-based Gene Set Analysis Toolkit (WebGestaltR)’ R package, we carried out the Gene Set Enrichment Analysis (GSEA) on different gene sets (Liao et al., 2019). In order to study the pathways of numerous biological activities in different groups, the GSEA for pathway analysis was conducted. Here, all candidate gene sets in the Hallmark database (Liberzon et al., 2015) were subjected to gene set enrichment analysis, with false Discovery Rate (FDR) < 0.05 being considered as a significant enrichment. In addition, the ‘Gene Set Variation Analysis (GSVA)’ R package was introduced for performing single sample GSEA (ssGSEA) on the gene expression profile of HCC samples in the TCGA cohorts (Hänzelmann et al., 2013). Each sample’s score for various functions was equal to its matching ssGSEA score for each function.

### Creating a protein-protein interaction network

The Search Tool for the Retrieval of Interaction Gene/Proteins (STRING) (https://string-db.org/) database was employed to search for protein-protein interaction (PPI) among predicted and known proteins. STRING is a database with the largest number of species and interaction information data, including 2031 species, 13.8 million protein interactions, and 9.6 million proteins. Studying the interaction network between proteins helps in finding the core regulatory genes. After building the PPI network, the Cytoscape was used for visualization (Su et al., 2014).

### Construction of a risk model

The TCGA data set was categorized into two groups according to the ratio of Train: Test = 1:1. The differences of clinical features between train and test sets were examined by Fisher’s exact test. Then the genes in the train data set were subjected to the univariate Cox regression analysis. The least absolute shrinkage and selection operator (Lasso) is a compression estimation [15] that creates a penalty function to shape an advanced model through compressing coefficients and setting some coefficients to zero. This study used the ‘glmnet’ R package (Friedman et al., 2010) for performing the Lasso Cox regression. In addition, the stepwise multivariate regression analysis was used. The stepwise regression employed the Akaike information criterion (AIC). The stepAIC approach in the Modern Applied Statistics with S (MASS) package (Zhang, 2016) begins with a complicated model and successively eliminates a variable to decrease the AIC, with a smaller value indicating a better model, which highlights a high fitting of the model with fewer parameters.

The risk-related prognostic risk score (RS) of each sample was calculated with the following formula: RS = Σβi × Expi, Expi is the expression level of gene characteristics, and βi represents the Lasso Cox regression coefficient of the corresponding gene. RS-high and -low groups of patients were divided under the median value of the threshold. Prognosis analysis and significant difference was determined by Kaplan-Meier (KM) method and the Log-rank test, respectively. In addition, we employed the ‘timeROC’ R package (Blanche et al., 2013) for performing receiver operating characteristic (ROC) analysis on RS prognosis classification.

### Prediction of immunotherapy effect

The Tumor Immune Dysfunction and Exclusion (TIDE) algorithm (http://tide.dfci.harvard.edu/) (Jiang et al., 2018) verified the efficacy of immune microenvironment score (IMS) on predicting clinical response of HCC patients to immune checkpoint inhibitors (ICIs). TIDE algorithm uses gene expression profiles to estimate the reactivity of immune checkpoint blockade (ICB) based on 3 cell types that limit T cell infiltration in tumors, specifically, myeloid-derived suppressor cells (MDSCs), the M2 subtype of tumor-associated macrophages (TAM), two varied tumor immune escape strategies, including tumor-infiltrating cytotoxic T lymphocyte (CTL) dysfunction score and CTL immunosuppressive factor rejection score and tumor-associated fibroblasts (TAF). Higher TIDE score pointed to a greater immune escape probability, suggesting less benefits of HCC with such a status from taking immunotherapy. Subclass mapping method (Roh et al., 2017) was used to compare the similarity of the expression profiles between the test group and the immunotherapy group for estimating the sensitivity of the test group to immunotherapy.

### Drug sensitivity analysis

For estimating the RS of predicting molecular drug response, the half-maximal inhibitory concentration (IC50) of drugs was evaluated by using the “pRRophetic” R package (Geeleher et al., 2014) in accordance with the expression profile in different data sets.

## Results

### Identification and functional analysis of the genes associated with HBV-related HCC

**FIGURE 1:**
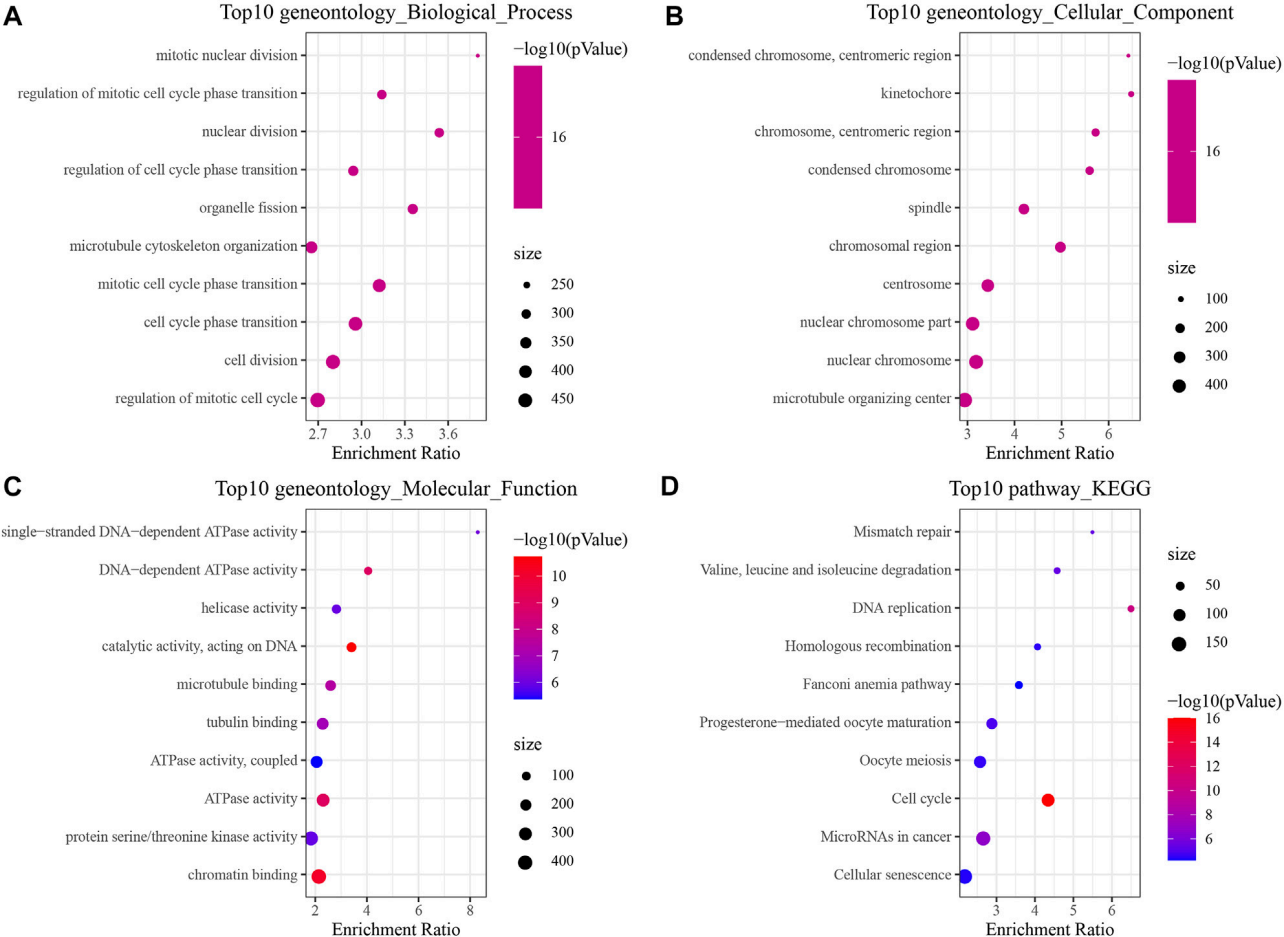
The results of functional enrichment analysis of HCCDB18. **(A)** BP annotation map of DEGs between HBV and non-HBV patients; **(B)** CC annotation map of DEGs between HBV and non-HBV patients; **(C)** MF annotation map of DEGs between HBV infected and non-HBV infected patients; **(D)** KEGG annotation map of DEGs between HBV and non-HBV patients.

In order to identify the DEGs in HBV-related HCC, we analyzed the variations between HBV and non-HBV patients in the HCCDB18 data set. Finally, 1344 DEGs were obtained, with 1168 of them upregulated and 176 genes downregulated in HBV patients. Then, these 1344 DEGs were assessed by the KEGG pathway and GO functional enrichment. 577 items with considerable differences in Biological Process (BP) were annotated for GO function of DEGs (Figure 1A, *p* < 0.05), including the processes of regulation of mitotic cell cycle phase, mitotic nuclear division, nuclear division, transition, mediation of cell cycle phase transition, and other items related to cell division. 144 items with significant difference in Cellular Component (CC) were annotated (Figure 1B, *p* < 0.05), including centromeric region, condensed chromosome, kinetochore, centromeric region, chromosome, and other chromosome-related items. We annotated 125 items with major differences in Molecular Function (MF) (Figure 1C, *p* < 0.05), including helicase activity, single-stranded DNA-dependent ATPase activity, catalytic activity, DNA-dependent ATPase activity, acting on DNA, and other ATPase enzymes. For the enrichment of the KEGG pathway of DEGs, 32 items were significantly annotated (Figure 1D, *p* < 0.05). Among them, homologous recombination, cell cycle, mismatch repair, DNA replication, microRNAs in cancer, and other pathways were also significant.

### PPI analysis of HBV gene in HCC

**FIGURE 2:**
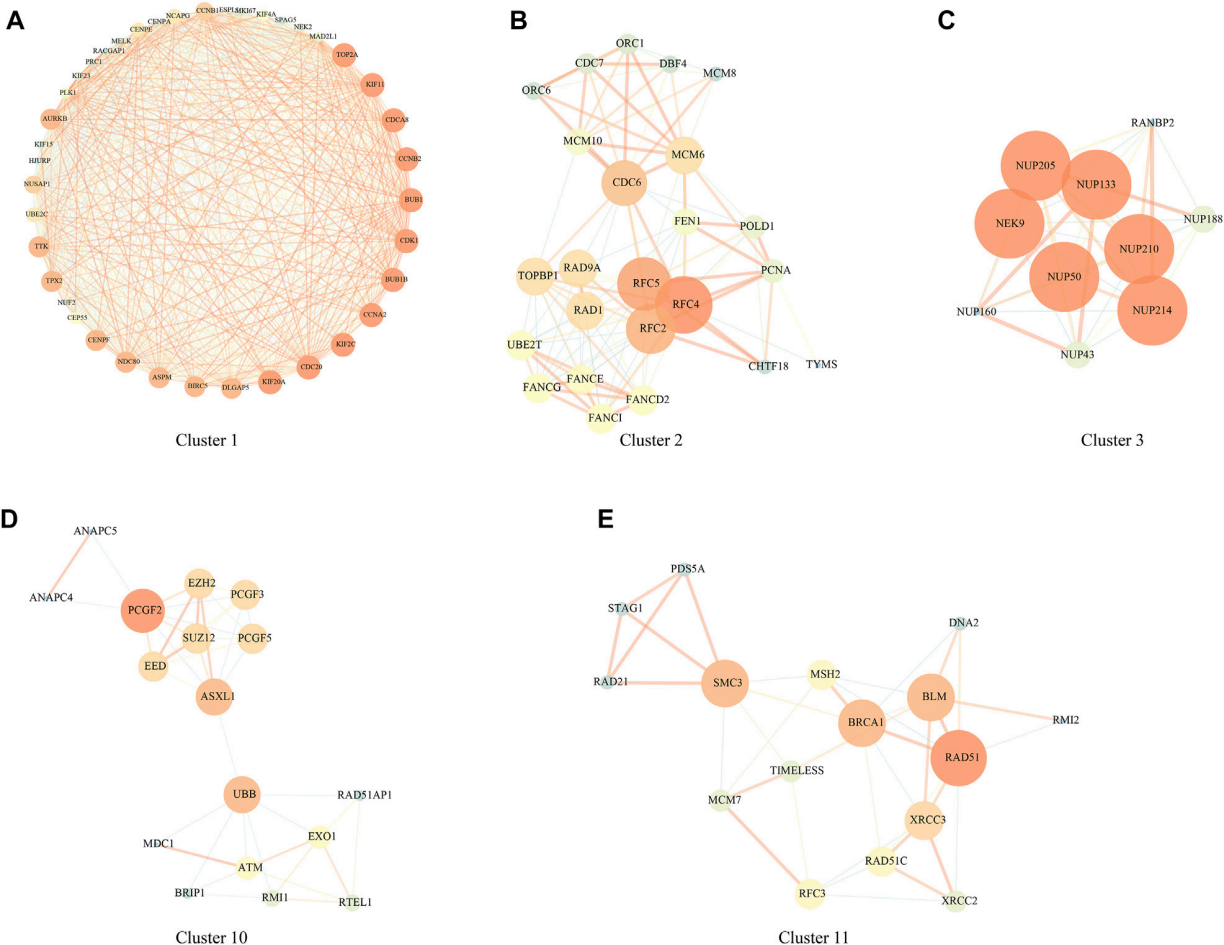
PPI network module results. **(A)** Cluster one network PPI analysis; **(B)** Cluster two network PPI analysis; **(C)** Cluster three network PPI analysis; **(D)** Cluster 10 network PPI analysis; **(E)** Cluster 11 network PPI analysis.

PPI analysis was carried out based on 1344 DEGs in the above HCCDB18 data set, and MCODE was used to find network function modules. Modules containing at least 10 genes were retained, including Cluster 1 (Figure 2A), Cluster 2 (Figure 2B), Cluster 3 (Figure 2C), Cluster 10 (Figure 2D), and Cluster 11 (Figure 2E).

The genes in the clusters were performed with KEGG pathway analysis and GO function enrichment analysis. Specifically, Cluster one was closely associated with the FoxO signaling pathway, Human T-cell leukemia virus one infection, p53 signaling pathway, and other pathways (Supplementary Figure S1); Cluster two was closely related to DNA replication, Mismatch repair, Homologous recombination, Base excision repair, and other pathways (Supplementary Figure S2); Cluster three was closely related to RNA transport, structural constituent nuclear pore and other pathways (Supplementary Figure S3); Cluster 10 was closely related to Homologous recombination, Fanconi anemia pathway, Cell cycle and other pathways (Supplementary Figure S4); Cluster 11 was closely related to homologous recombination, mismatch repair, DNA replication, cell cycle and other pathways (Supplementary Figure S5);

### Construction of a prognostic model related to HBV in HCC

**FIGURE 3:**
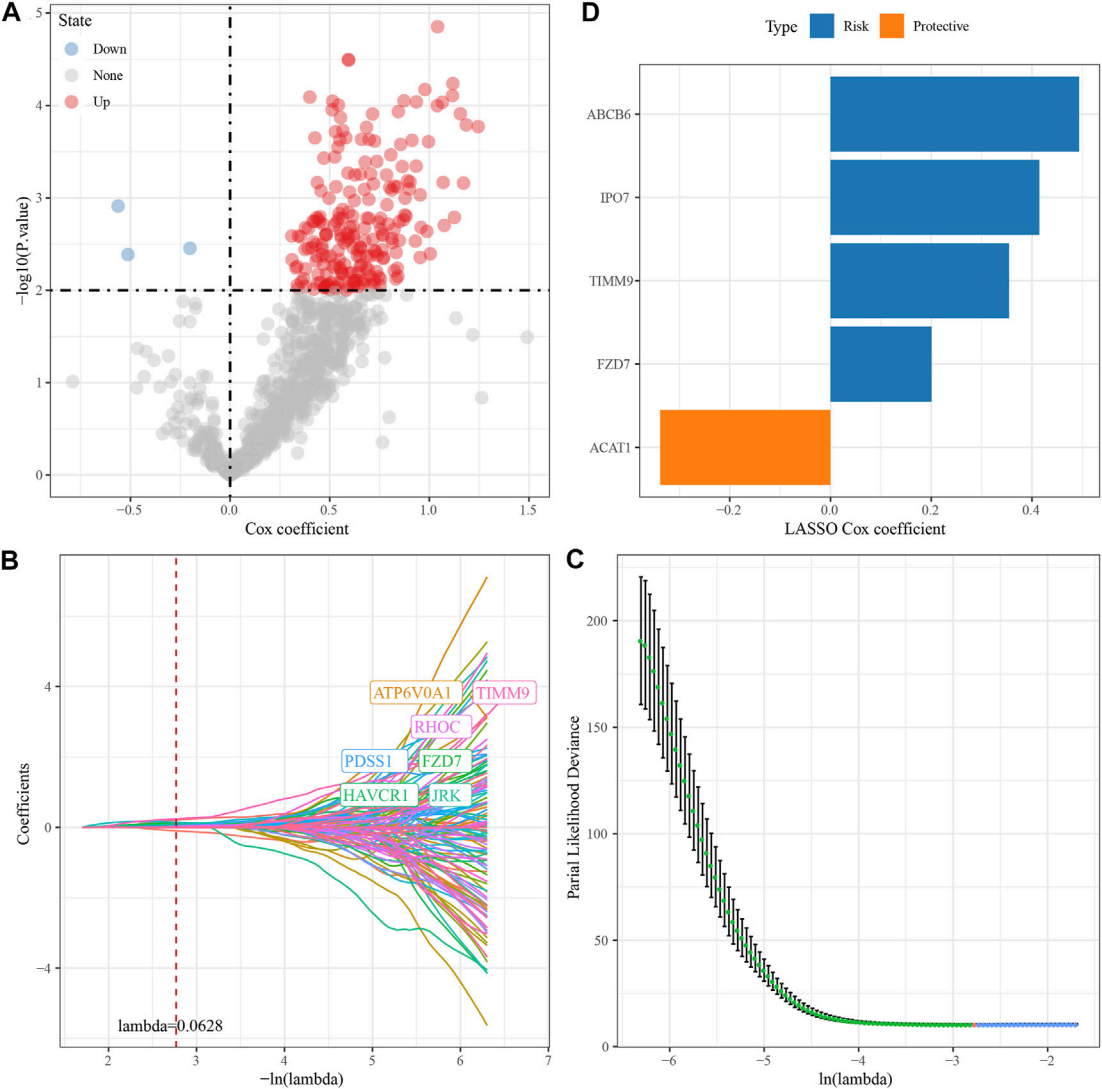
Construction of HBV gene prognostic model for HCC. **(A)** Analysis results of DEGs; **(B)** The locus of each independent variable changing with lambda; **(C)** CI under lambda; **(D)** Lasso coefficient distribution of HBV-related gene characteristics.

We randomly divided TCGA dataset into train and test data sets and there was no significant difference of their clinical features between two data sets (Supplementary Table S1). For 472 HBV-related DEGs in the PPI network, univariate Cox regression analysis was conducted using the Train data set in the TCGA data set. Finally, a total of 222 genes, including 219 “Risk” and 3 “Protective” genes (*p* < 0.01) with great impact on prognosis, were identified (Figure 3A). The 222 genes in the Train data set were further compressed by Lasso regression to reduce the genes in the risk model. We assessed the change trajectory of individual independent variables and discovered that a mutual increase between number of independent variable coefficients tending to 0 and lambda. The model reached its optimum efficiency at a lambda value of 0.0628 (Figure 3B). Then, a model was developed by performing 10-fold cross-validation, and confidence interval under each lambda was analyzed (Figure 3C). Finally, 14 genes with lambda = 0.0628 were chosen as the target genes. Based on the 14 genes in Lasso analysis, five genes (ABCB6, IPO7, TIMM9, FZD7, and ACAT1) were identified as HBV-related genes affecting prognosis by stepwise multivariate regression analysis (Figure 3D). The prognostic model was defined as risk score = 0.494*ABCB6 + 0.355*TIMM9 + 0.201*FZD7 + 0.415*IPO7—0.338*ACAT1.

### Development and validation of the clinical prognostic model

**FIGURE 4:**
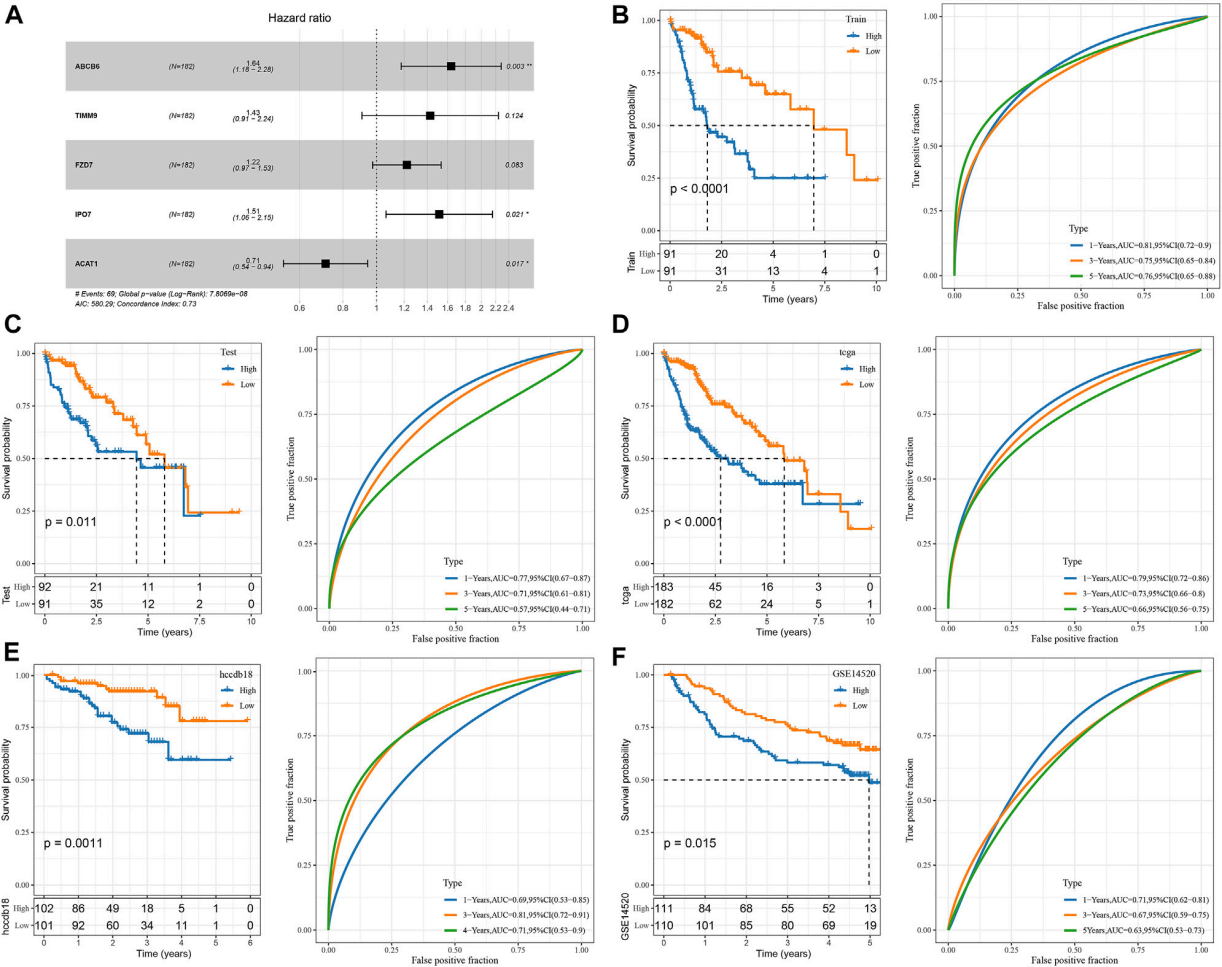
Construction and validation of the clinical prognostic model. **(A)** Multivariate Cox forest map of model genes; **(B)** ROC curve and KM survival curve of RS in TCGA training data cohort; **(C)** ROC curve and KM survival curve of RS in the TCGA validation data cohort; **(D)** ROC curve and KM survival curve of RS in TCGA cohort; **(E)** ROC curve and KM survival curve of RS in HCCDB18 cohort; **(F)** ROC curve and KM survival curve of RS in GSE14520 cohort.

The genes of the above clinical prognostic models were analyzed by multivariate analysis (Figure 4A). Moreover, ROC analysis of prognosis classification was carried out based on the RS of each sample. We assessed the grouping efficiency of one-, three- and 5-year prognosis prediction of the training data set (Figure 4B). The area under the ROC curve (AUC) values were 0.81, 0.75, and 0.76, respectively. Finally, with the median value as the cutoff, we sorted the samples into RS-high and -low groups and drew the KM curve. It can be observed that there is a substantial variation between RS-low and -high groups (*p* < 0.0001). Number of samples in both the RS-high group and RS-low group was 91. Patients with higher RS showed worse overall survival in the training cohorts. To confirm the robustness of risk-related genes in the prediction of the clinical prognostic model, we verified them in the TCGA validation data set (Figure 4C) and TCGA all data set cohort (Figure 4D). Patients’ RS was similarly measured. The validation cohorts showed similar results to the training sets. High RS had a poor prognosis, while low RS was the opposite. Simultaneously, we performed verification in the independent data sets HCCDB18 (Figure 4E) and GSE14520 (Figure 4F). The validation cohorts showed the same outcomes as the training set, proving the reliability of our results. Compared with other prognostic models of HCC from Zheng et al. (4-gene signature) (Zheng et al., 2018), Hu et al. (7-gene signature) (Hu et al., 2020), Ke et al. (6-gene signature) (Ke et al., 2018), and Liu et al. (3-gene signature) (Liu et al., 2019), our model showed a relatively higher AUC in predicting 1-year and 3-year survival (Supplementary Figure S6).

**FIGURE 5:**
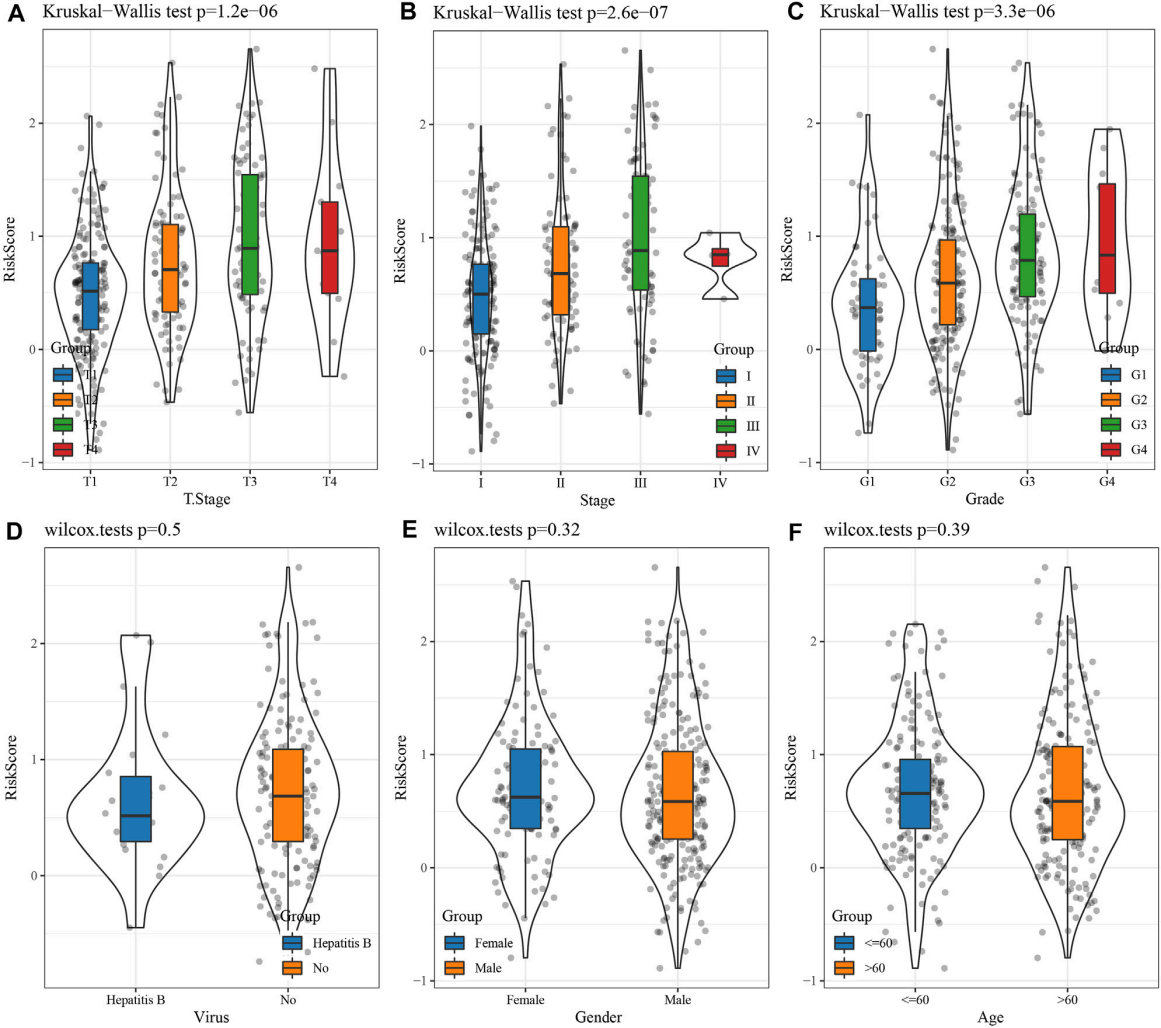
Differences in RSs among different clinicopathological groups in the TCGA cohort. **(A)** T Stage; **(B)** Stage; **(C)** Grade; **(D)** Virus; **(E)** Gender; **(F)** Age. (ns, *p* > 0.05; * * *, *p* < 0.001; * *, *p* < 0.01; *, *p* < 0.05).

Comparison of the RS distribution among clinical-pathological features groups demonstrated a major variation in RS among T stage, stage, grade, *etc.* In TCGA data set (Figures 5A, B, C). In T Stage, the RS of the T1 Stage was the lowest (Figure 5A). No major variations were observed in RS in relation to virus, gender, or age (Figures 5D, E, F).

### Mutation characteristics between RS groups

**FIGURE 6:**
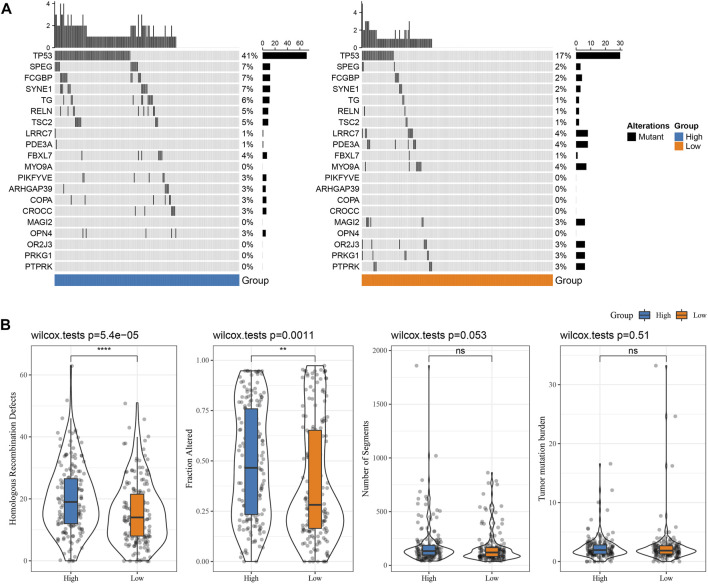
Genome changes of RS groups in TCGA cohort. **(A)** Somatic mutation analysis of various RS groups in TCGA cohort (fisher’s exact test); **(B)** Differences in Homologous Recombination Defects, Fraction Altered, Number of Segments, and Tumor mutation burden in different RS groups of TCGA cohort. (ns, *p* > 0.05; * * *, *p* < 0.001; * *, *p* < 0.01; *, *p* < 0.05).

The differences in genome changes among different RS groups in the TCGA cohort were discussed. Therefore, we analyzed the mutation characteristics of 37 high-frequency mutant genes in different groups. It has been found that the mutation frequency of TP53 in the RS-low group (41%) was increased than the RS-high group (17%), and that the mutation frequency of SPEG in the RS-low group (7%) was increased than the RS-high group (2%), while that of LRRC7 in the RS-low group (1%) was lower when compared than the RS-high group (4%) (Figure 6A). Further distribution comparison of fraction altered, tumor mutation burden, homologous recombination defects, and the number of segments among different groups demonstrated that fraction altered and homologous recombination defects scored considerably higher in the RS-high group than those in the RS-low group (Figure 6B).

### Pathways characteristics between RS groups

**FIGURE 7:**
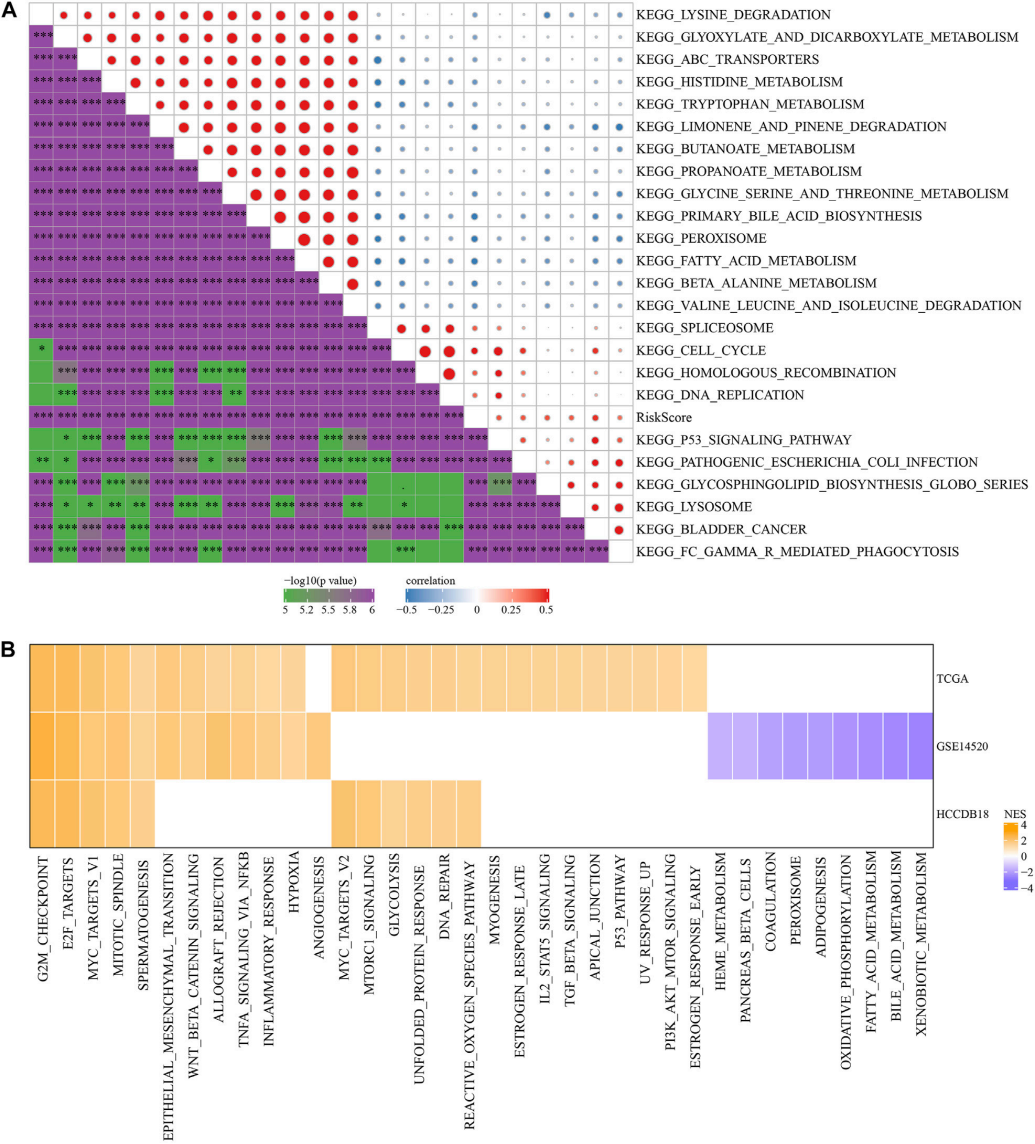
Pathway characteristics between RS groups. **(A)** The correlation analysis results between the KEGG pathway and RS whose correlation with RS in TCGA cohort is greater than 0.35; **(B)** RS-high and RS-low enrichment fractional heat maps.

To investigate the association of RS with the biological role of different samples, we further investigated the correlation between these functions and RS and determined functional pathways with a correlation greater than 0.35 (Figure 7A). These pathways were positively correlated with RS of samples and were mainly tumor-related (KEGG_HOMOLOGOUS_RECOMBINATION, KEGG_DNA_REPLICATION, KEGG_P53_SIGNALING_PATHWAY, KEGG_BLADDER_CANCER). Simultaneously, it was negatively correlated with metabolic pathways, such as KEGG_FATTY_ACID_METABOLISM, KEGG_HISTIDINE_METABOLISM, KEGG_TRYPTOPHAN_METABOLISM, *etc.*

Next, we analyzed whether there were differentially activated pathways in different RS groups. Compared with the RS-low group in TCGA cohort, 26 pathways in the RS-high group were activated, 12 pathways in the GSE14510 cohort were activated, nine pathways were inhibited, and 11 pathways in the HCCDB18 cohort were activated. On the whole, the activated pathways in the RS-high group were mainly tumor-related pathways such as EPITHELIAL_MESENCHYMAL_TRANSITION, MYC_TARGETS_V1, TNFA_SIGNALING_VIA_NFKB, and G2M_CHECKPOINT, *etc.* (Figure 7B, False Discovery Rate (FDR) < 0.05).

### The difference in immunotherapy/chemotherapy among groups

**FIGURE 8:**
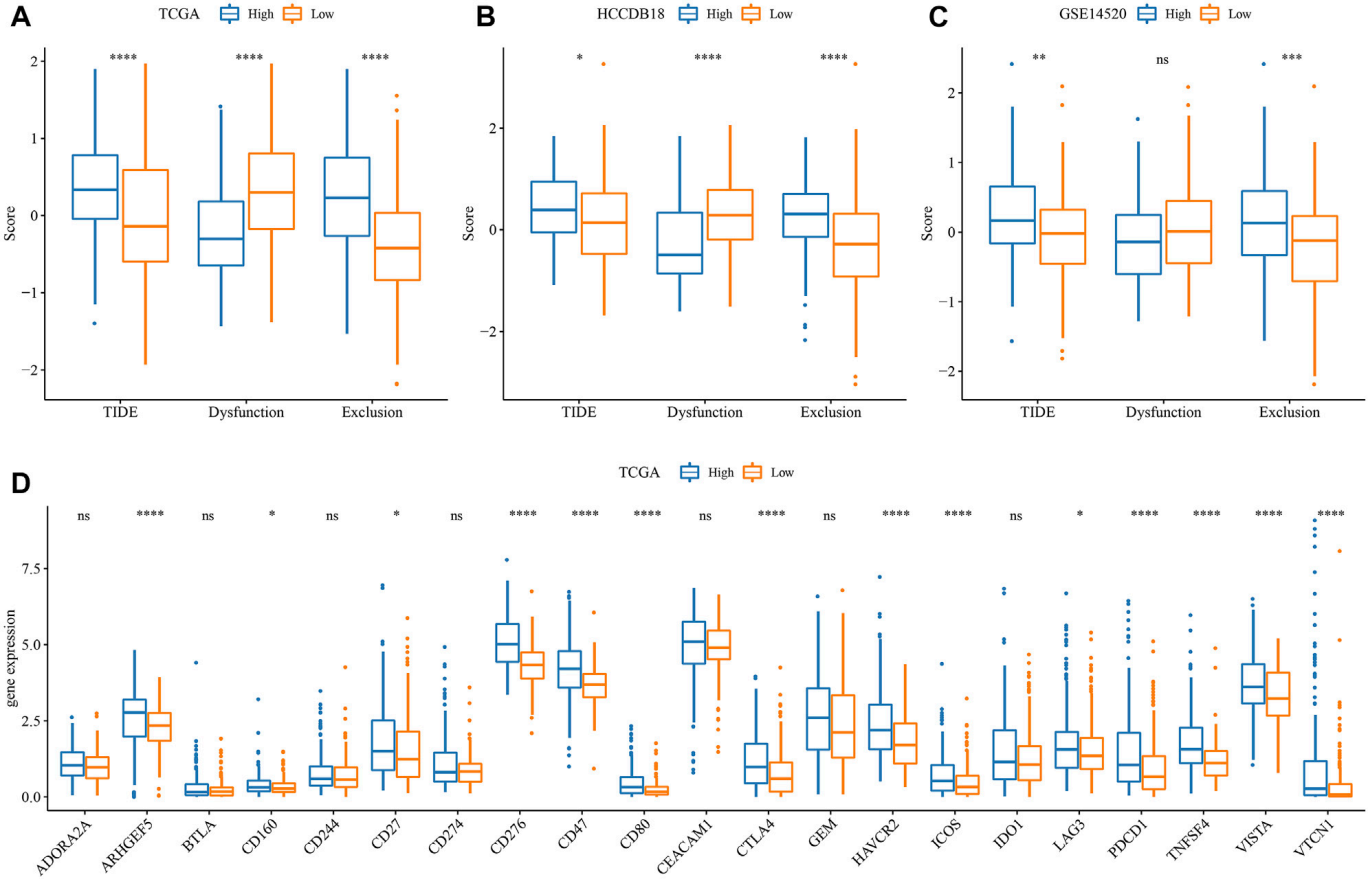
Difference analysis of immunotherapy. **(A)** Differences in the results of TIDE analysis among different groups in TCGA cohort; **(B)** Differences in TIDE analysis results among different groups in HCCDB18 queue; **(C)** Differences in TIDE analysis results among different groups in GSE14520 queue; **(D)** Immune checkpoints differentially expressed between different groups in the TCGA cohort. (ns, *p* > 0.05; * * *, *p* < 0.001; * *, *p* < 0.01; *, *p* < 0.05).

First, the differences in immunotherapy in different groups were analyzed, and TIDE was employed for analyzing clinical effect of immunotherapy in our described RS-low and -high groups. In the TCGA (Figure 8A), HCCDB18 (Figure 8B), and GSE14520 (Figure 8C) cohorts, the TIDE score in the RS-high group was much higher than that in the RS-low group, and was consistent in different data sets, suggesting that the RS-high group had increased possibility of immune escape and less benefit from taking immunotherapy. In addition, comparison on the expression of immune checkpoints among groups was conducted. Here, our immune checkpoints were provided by HisgAtlas (Liu et al., 2017). It could be seen that some immune checkpoint genes were differentially expressed in TCGA data set (Figure 8D).

**FIGURE 9:**
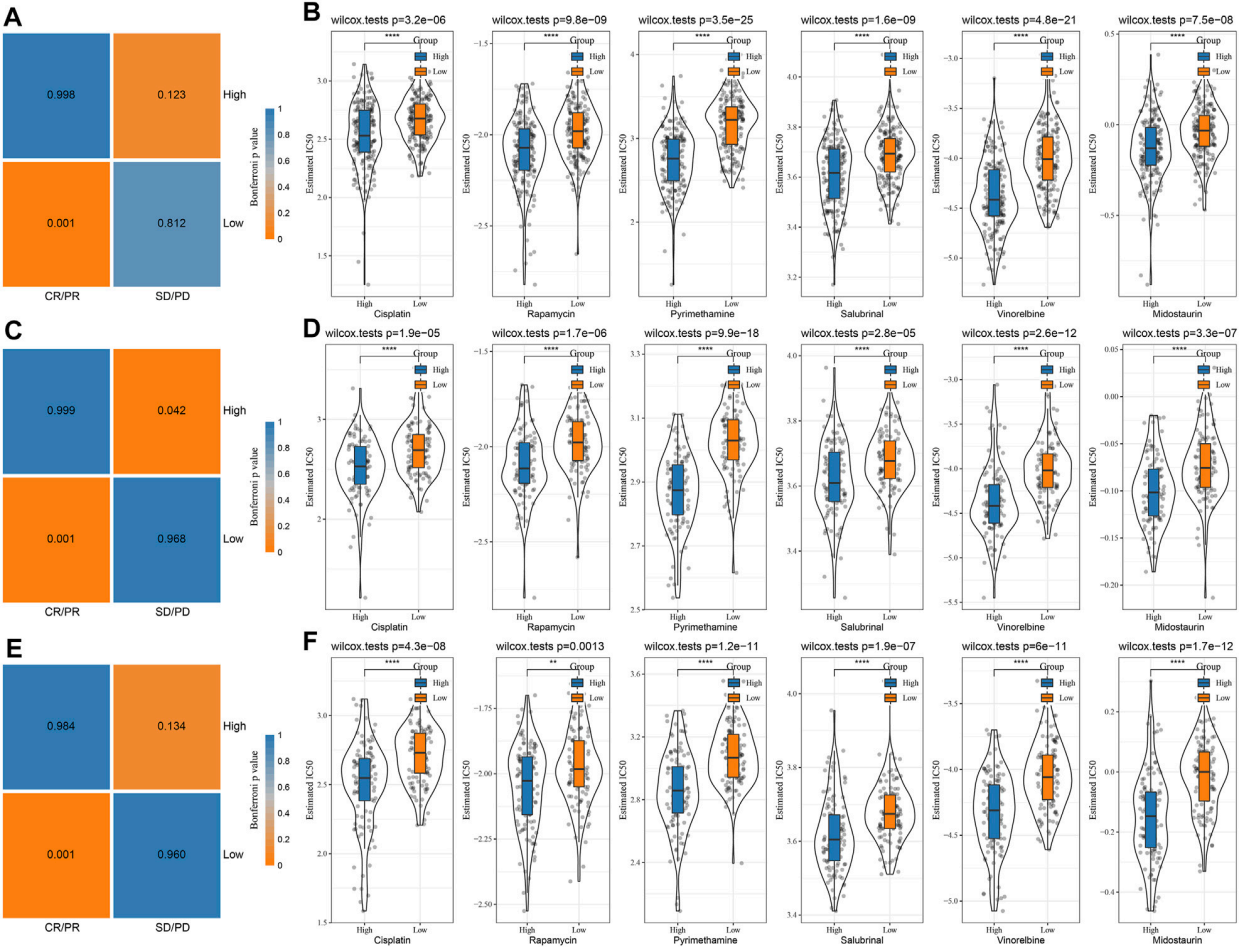
Immunotherapy mapping and drug sensitivity analysis. **(A)** Immunotherapy mapping of different risk groups of TCGA; **(B)** Estimated IC50 box diagram of cisplatin, rapamycin, pyrimethamine, salubrinal, vinorelbine, and midostaurin in TCGA; **(C)** Immunotherapy mapping of different risk groups of HCCDB18; **(D)** Estimated IC50 box diagram of cisplatin, rapamycin, pyrimethamine, salubrinal, vinorelbine and midostaurin in HCCDB18; **(E)** Immunotherapy mapping of different risk groups of GSE14520; **(F)** Estimated IC50 box diagram of cisplatin, rapamycin, pyrimethamine, salubrinal, vinorelbine and midostaurin in GSE14520. (ns, *p* > 0.05; * * *, *p* < 0.001; * *, *p* < 0.01; *, *p* < 0.05).

The differences between chemotherapy and immunotherapy in different immune molecular subtypes were analyzed. Here, we employed the subclass mapping method (Roh et al., 2017) for comparing the similarity between the risk groups in our defined data sets and the immunotherapy patients in IMvigor210 data sets. A reduced *p*-value indicated increased similarity. The results showed that in TCGA (Figure 9A), HCCDB18 (Figure 9C), and GSE14520 (Figure 9E) data sets, the RS-low group was more sensitive to programmed cell death-Ligand 1 (PD-L1) treatment, while the RS-high group might not be sensitive to the treatment of PD-L1. This was consistent with the result of TIDE.

In addition, the analysis of the responsiveness of the TCGA (Figure 9B), HCCDB18 (Figure 9D), and GSE14520 (Figure 9F) cohorts to the traditional chemotherapy drugs cisplatin, rapamycin, pyrimethamine, salubrinal, vinorelbine, and midostaurin showed that the RS-high group was more sensitive to the mentioned drugs.

### Improvement of a prognostic model and survival prediction by RS combined with clinicopathological characteristics

**FIGURE 10:**
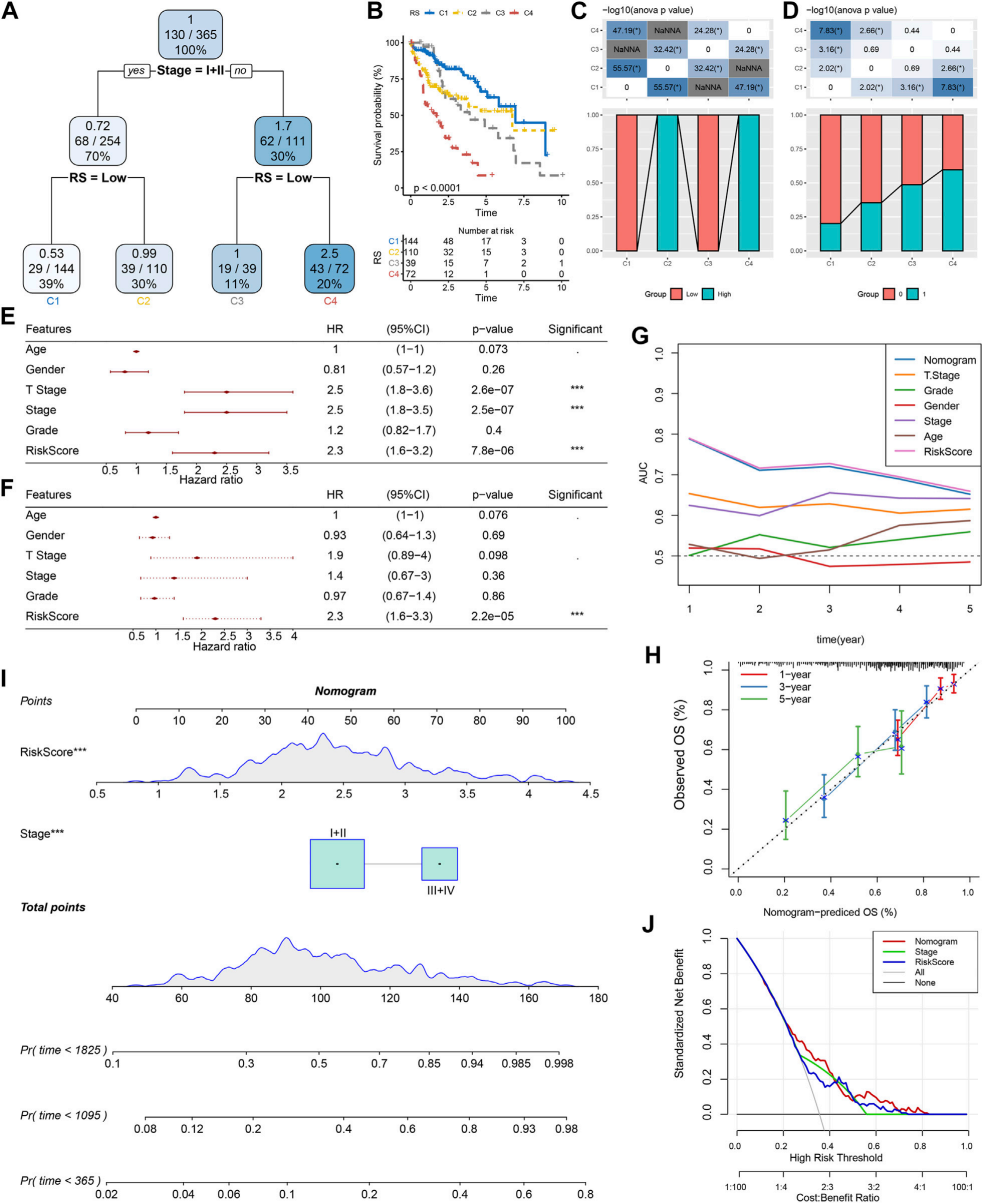
Improvement of a prognostic model and survival prediction. **(A)** The survival decision tree was constructed by using all annotations of patients, including RS, stage, gender, and age, to optimize risk stratification; **(B)** Overall survival analysis of three risk subgroups; **(C–D)**: Comparative analysis between different groups; E–F: univariate and multivariate Cox analysis of RS and clinicopathological characteristics; **(G)** Nomograph model; **(H)** Calibration curve of nomograph in 1, 3 and 5 years; **(I)** ROC curves of different clinicopathological characteristics at different times; **(J)** Decision curve of nomograph.

According to the sex, T Stage, Stage, grade, age, stage, and RS of HCC patients in the TCGA cohort, a decision tree was generated. The outcomes revealed that only RS and Stage were left in the decision tree, and four different risk subgroups were identified (Figure 10A). Stage and RS were the most powerful parameters. Major variations were observed in the overall survival among the four risk subgroups, of which C1 had the highest survival rate and C4 had the lowest (Figure 10B). Risk subgroups C2 and C4 were RS-high patients, while patients in groups C1 and C3 were RS-low patients (Figure 10C). Moreover, the survival status of patients in different risk subgroups was different (Figure 10D). Univariate and multivariate Cox regression analysis of clinicopathological properties and RS confirmed the later one as the most significant prognostic factor (Figures 10E, F). In univariate Cox regression analysis, the hazard ratio (HR) value of RS was 2.3, the 95% confidence interval (CI) was 1.6–3.2, and the *p*-value was 7.8e-06 (Figure 10E), while in multivariate regression analysis, the HR value of RS was 2.3, 95%CI was 1.6–3.3, and *p*-value was 2.2e-05 (Figure 10F). A nomogram (Figure 10G) was established in combination with RS and other clinicopathological characteristics to quantify the risk assessment and survival probability of patients with HCC. From the model results, RS impacted the survival prediction the most. The calibration curve was applied for evaluating the model’s prediction accuracy (Figure 10H). The nomogram had strong prediction performance because the anticipated calibration curve for the three calibration points in 1, 3, and 5 years was near to the standard curve. In addition, to investigate the model’s reliability, decision curve analysis (DCA) was utilized. And we found that the accuracy of using RS and nomogram was considerably higher in comparison with those of the extreme curve. The nomogram and RS showed the strongest ability to predict survival (Figures 10I, J) in compared with other clinicopathological characteristics.

### Construction of a diagnostic model of HBV gene in HCC

**FIGURE 11:**
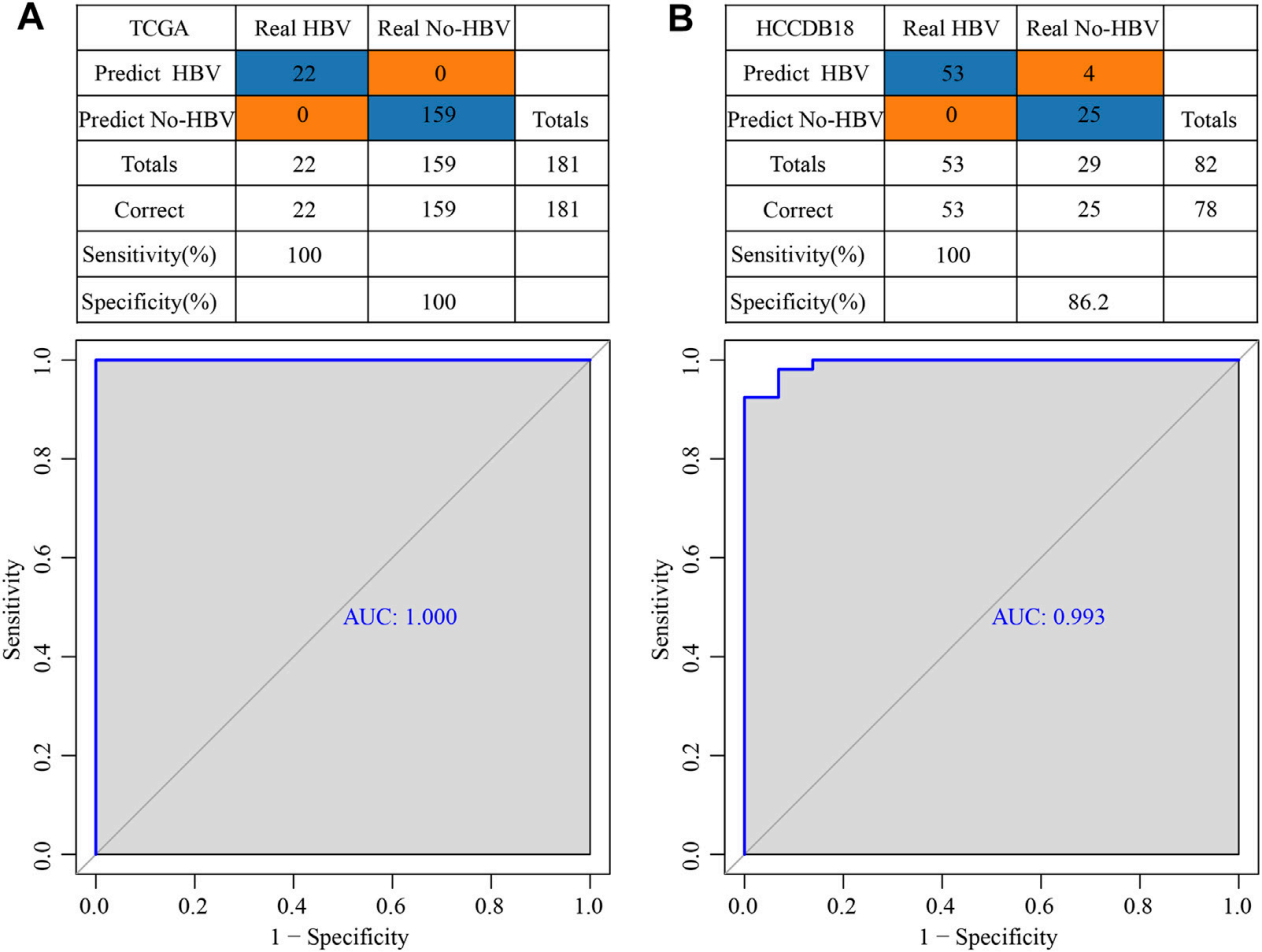
Construction of a diagnostic model of HBV gene in HCC. **(A)** The classification outcomes and ROC curves of samples in TCGA by diagnostic model; **(B)** The classification outcomes and ROC curves of samples in HCCDB18 samples by diagnostic model.

TCGA was used as the training data set, and for the validation dataset we used HCCDB18. In the training data set, five genes of the prognostic model were characterized to obtain their corresponding expression profiles. A support vector machine (SVM) was constructed to distinguish HBV patients from non-HBV patients. The classification accuracy was 100%, and 181 samples were classified appropriately. The model’s sensitivity and specificity were 100%, and the value of AUC was 1 (Figure 11A). The HCCDB18 data set was used to verify that 78 of 82 samples could be classified accurately. The model’s sensitivity was 100%, its specificity was 86.2%, and its AUC was 0.993. The classification accuracy was 95.12% (Figure 11B). These findings demonstrated that the diagnostic and prognostic models developed in this study were capable of accurately differentiating HBV patients from non-HBV patients with HCC, and that the five genes identified here can serve as reliable biomarkers for the diagnosis of HCC.

## Discussion

Although the clinical therapy of HCC and our understanding of its pathophysiology have been significantly advanced, the incidence rate and mortality of this malignant tumor remain noticeably high. In China and other parts of Asia, chronic hepatitis B is the major cause of HCC (Yang et al., 2012). Thus, it is important to construct the prognostic and diagnostic models of HBV gene-related HCC. Public databases such as TCGA and GEO store massive data sets of high-throughput sequencing technologies, for instance, chips and RNA-seq, which enable us to carry out integrated data mining and overcome the limitations of small sample size in a single cohort and heterogeneity among samples. In this report, firstly, 1344 DEGs in total were identified between HBV and non-HBV patients in tumor patients, including 1168 high-expressed genes and 176 down-regulated genes. These 1344 DEGs were considerably enriched in cell division activities, according to GO analysis (such as nuclear division, regulation of mitotic cell cycle transition, regulation of nuclear division, and cell cycle transition). KEGG pathway analysis showed that mismatch repair, DNA replication, homologous recombination, cell cycle, microRNA, and other pathways were significant in tumors. These enrichment analysis results confirmed the mentioned outcomes were consistent with prior research, in which that HBV-related genes in HCC were found to be related to cell division, DNA replication, and other functions (Zeng et al., 2019; Wu et al., 2021).

After that, five HBV-related genes, ABCB6, IPO7, TIMM9, FZD7, and ACAT1, which affect the prognosis, were identified by multiple strategy combinations. It has been reported that ABCB6 is one of the biomarker genes capable of effectively predicting the clinical diagnosis, prognosis, and immune microenvironment of HCC with ferroptosis and iron metabolism characteristics (Tang et al., 2020). Furthermore, it has been found that the biomarkers ABCB6 DNA methylation and mRNA levels can be utilized for predicting the early intrahepatic recurrence of HCC caused by the hepatitis C virus (Tsunedomi et al., 2013). Meanwhile, it has been reported that IPO7 can combine with MTBP to participate in the regulatory mechanism of HCC metastasis (Ranjan et al., 2018). In addition, research has shown that FZD7 is up-regulated in gastric cancer, esophageal cancer, and HCC (Katoh and Katoh, 2005), and is the target gene of tumor-suppressive miRNA miR-504. FZD7 can stimulate the proliferation and invasion of HCC cells through Wnt/β-catenin signal transduction (Quan et al., 2018). Other reports have highlighted that the down-regulation of ACAT1 is substantially linked with a poor prognosis of HCC patients who have elevated HbA1c (Bi et al., 2021). And some literature has shown that ACAT1-mediated acetylation of GNPAT to stabilize FASN plays a key role in hepatocarcinogenesis (Gu et al., 2020). Four of these five genes were reported previously as oncogenes, therapeutic targets, or useful biomarkers in HCC, which fully confirmed the reliability of our analysis results.

In addition, it has been reported that FZD7 can promote the tumor development of HCC cells *in vivo via* Wnt/β-catenin signal transduction in HBV-induced HCC (Kim et al., 2008). The other four prognostic genes have not been shown to be associated with HBV in reports. Therefore, this study was the first proposed the relationship between ABCB6, IPO7, TIMM9, and ACAT1 and HBV, and they may be related to tumor progression in HBV-induced HCC as FZD7. In addition, this study has established a clinical prognostic model and categorized RS-high and -low groups. We continued to focus on whether the efficacy of immunotherapy was different among groups. Previous research has demonstrated that HCC can cause an immunosuppressive tumor immune milieu and accelerate the growth and spread of tumors in various ways (Shuai et al., 2016). Immunotherapies, like immune checkpoint inhibitors, have been reported to have efficacious antitumor activity. Although only a few patients respond to immunotherapy (Ruiz de Galarreta et al., 2019; Wang and Wang, 2019), our analysis showed that some immune checkpoint genes were differentially expressed. Because PD-L1 can mediate immune escape of hepatoma tumor cells (Gao et al., 2018; Yan et al., 2020), follow-up analysis was also conducted. The analysis of immunotherapy and chemotherapy showed that the RS-low group was more sensitive to PD-L1 treatment, while the RS-high group may not show sensitivity to PD-L1 treatment. Therefore, patients having low RSs may be more responsive to immunotherapy.

HBV infection will have complex biological impacts on the tumor microenvironment, which could partially reduce the effectiveness of immunotherapy (Li et al., 2020). HCC is known to be a highly heterogeneous disease that has different immune microenvironments between tumors and surrounding tissues (Chen et al., 2016). Chronic inflammation is usually known to be the continuous expression of different cytokines and the adding of immune cells to the diseased areas (Makarova-Rusher et al., 2015). Immunosuppression is stimulated by HBV infection and then peripheral immune tolerance develops with the progress of chronic infection. Finally, it mediates tumorigenesis as a result of compromised immune surveillance (Vandeven and Nghiem, 2014). Immunosuppressive checkpoints, such as programmed death 1 (PD-1)/PD-L1, T cell immunoglobulin domain and mucin domain-3 (TIM-3), CTLA-4, play a significant role in immunosuppression in chronic viral hepatitis by suppressing T cell responses (Makarova-Rusher et al., 2015). HBV promotes some signaling pathways composed of PD-1/PD-L1. This explains to some extent that patients with low RSs may be more responsive to immunotherapy through the PD-L1 signaling pathway. Although this paper has performed sufficient analysis, our research still has several limitations. First, a larger cohort is needed for further validation of these outcomes. Secondly, a detailed study is required to further analyze the specific role of the chosen five HBV-related genes in affecting the prognosis in HCC through *in vitro* and *in vivo* tests. Thirdly, the particular interaction and regulation mechanism of related genes in the prognostic and diagnostic model should be studied in detail. To overcome the limitations of this study, we will re-collect and expand clinical samples in the follow-up work, and try to verify the accuracy of the models with more external experiments. For the verification of the effectiveness of the models in the timely diagnosis and treatment of HCC, large-scale independent research is required in the future.

The independent assessment of TCGA, HCCDB18, and GSE14520 data sets confirmed the reliability and effectiveness of our immunophenotypic analysis model. Through a series of analyses, we developed a prognostic and diagnostic model of HCC, contributing to the understanding of the prognostic characteristics of HBV-related HCC patients and providing novel insight and foundation for detailed investigation of individual differences in immunotherapy.

## Conclusion

The RS clinical prognostic model was constructed using to HBV-related genes. The model showed a strong robustness and was independent of clinical-pathological characteristics. In conclusion, this prognostic model had a high prediction accuracy and survival prediction ability. Finally, a diagnostic model was constructed based on the prognostic model.

## Data Availability

The original contributions presented in the study are included in the article/Supplementary Material, further inquiries can be directed to the corresponding authors.
