# Supplementary figures and images for: Construction of HBV gene-related prognostic and diagnostic models for hepatocellular carcinoma
Source: Front Genet. 2023 Jan 4;13:1065644. doi: 10.3389/fgene.2022.1065644 (PMC9845411; doi:10.3389/fgene.2022.1065644)

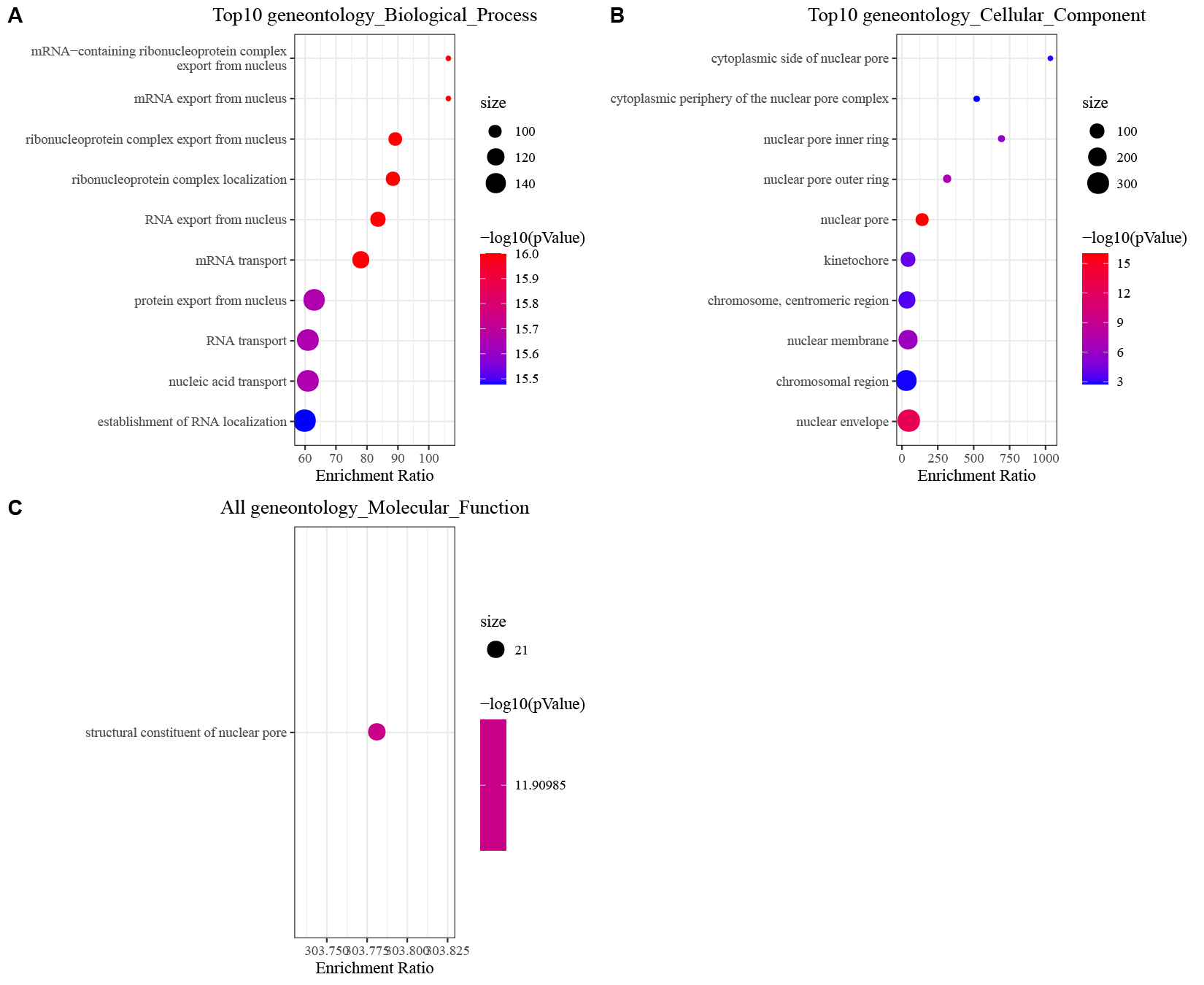

Supplement: Supplementary file 1 [file Image3.JPEG]

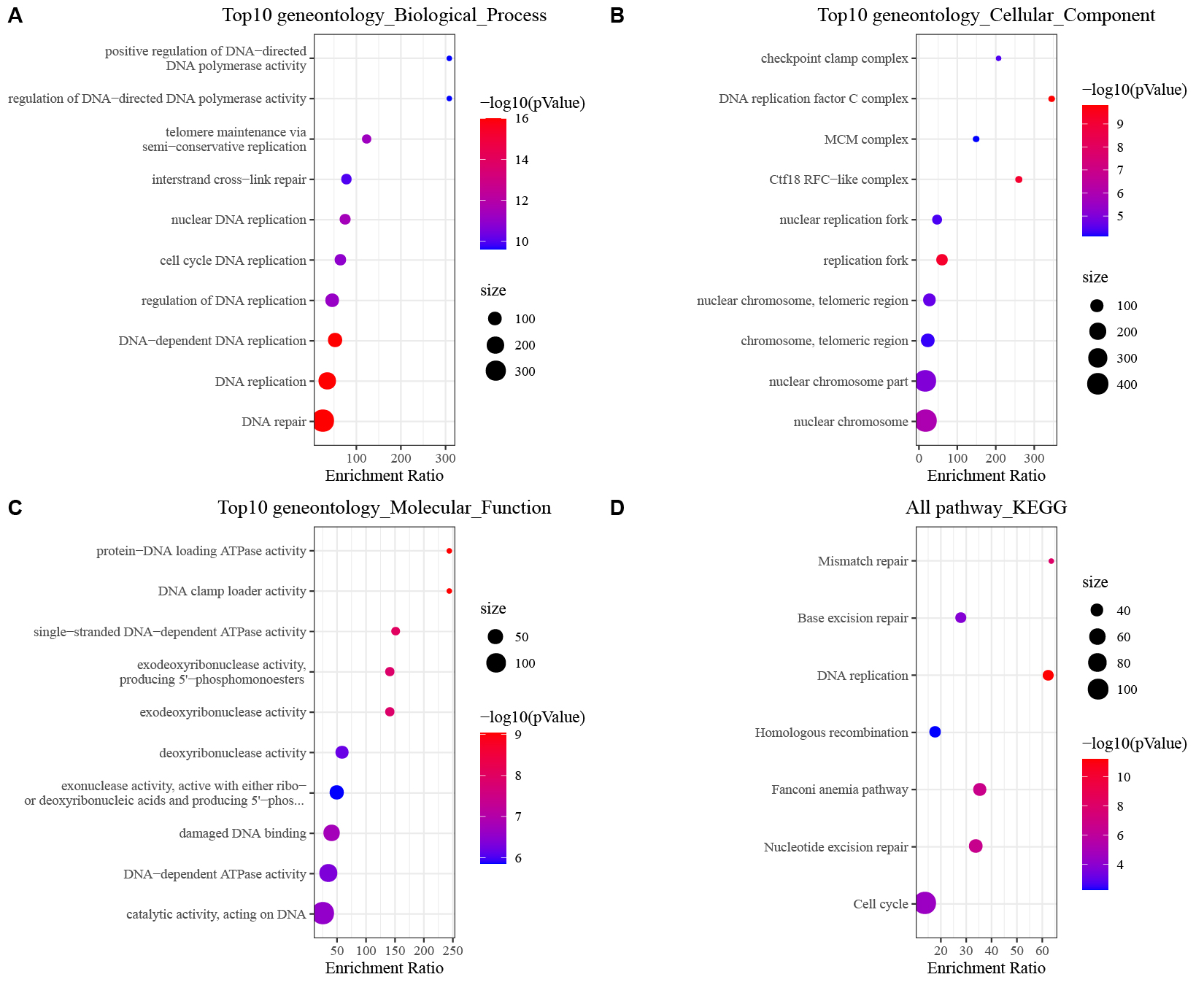

Supplement: Supplementary file 3 [file Image1.JPEG]

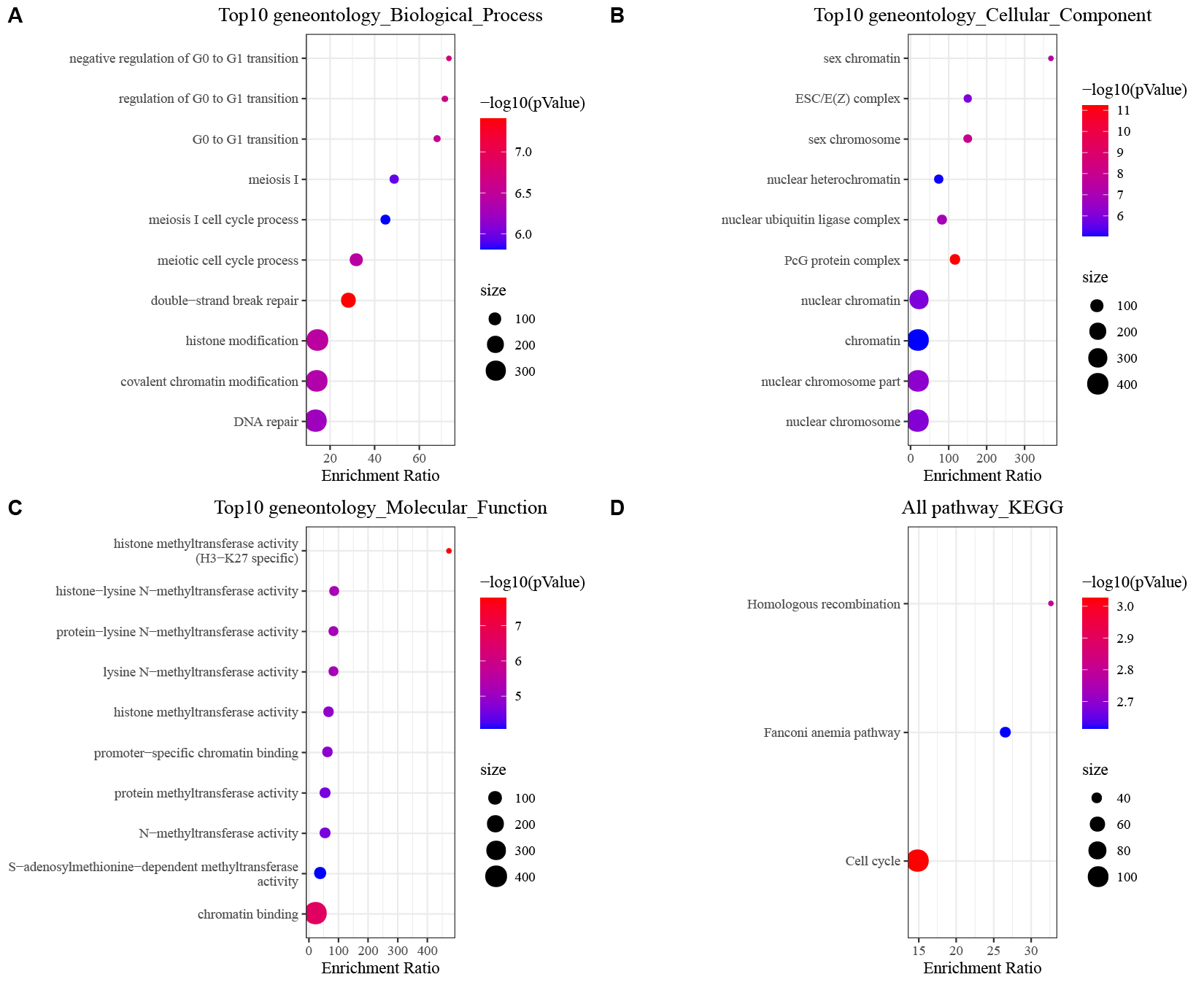

Supplement: Supplementary file 4 [file Image4.JPEG]

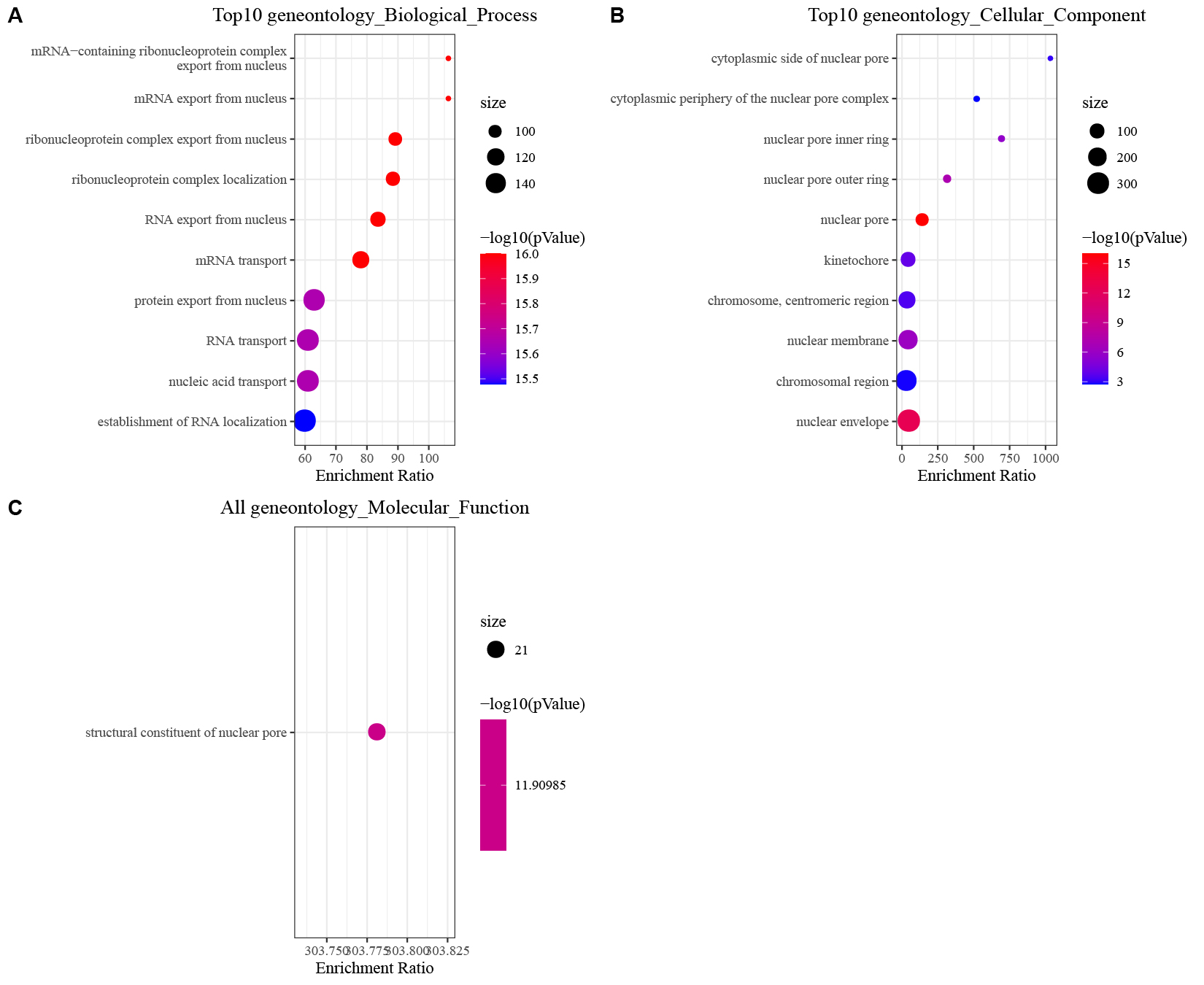

Supplement: Supplementary file 5 [file Image2.JPEG]

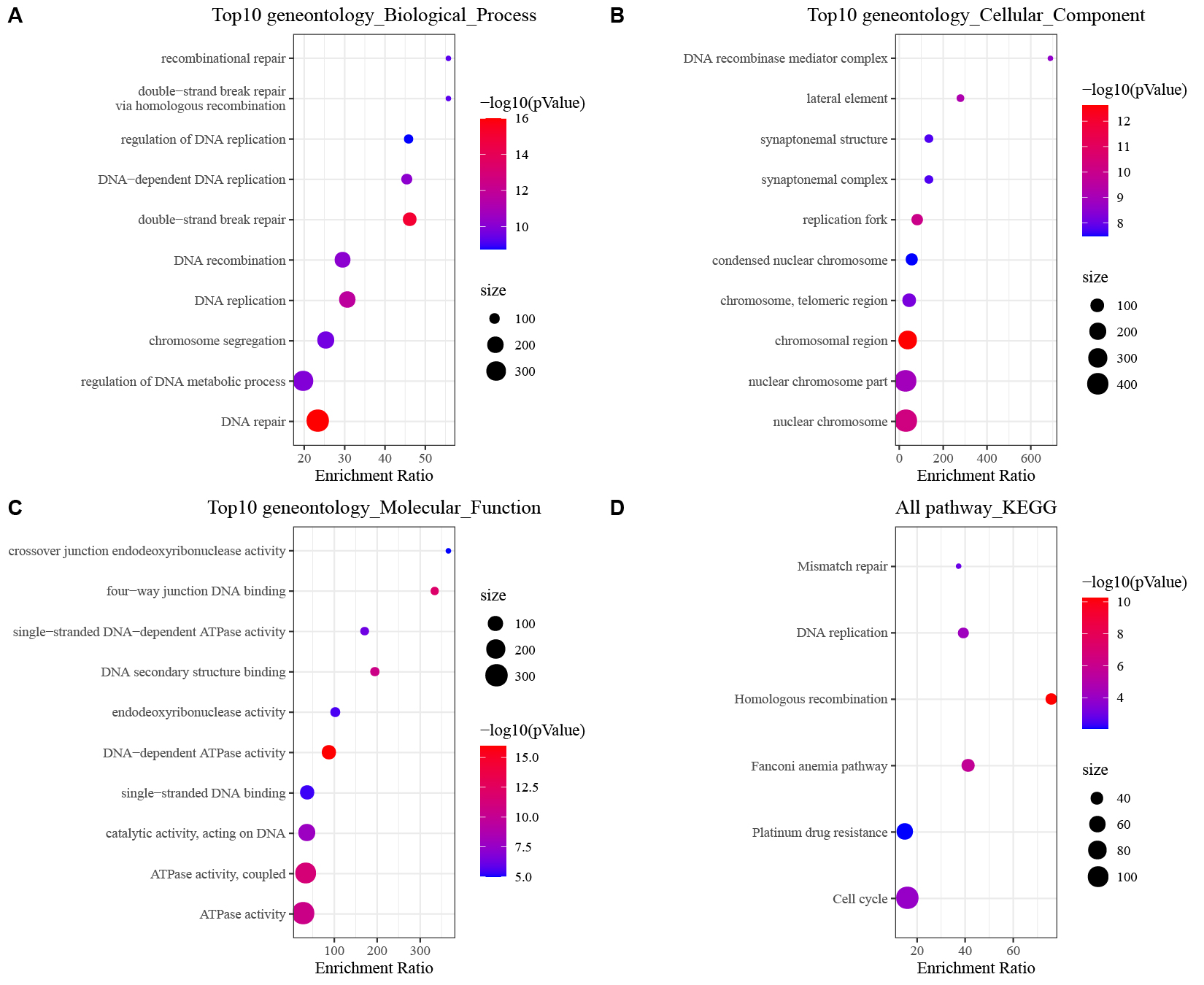

Supplement: Supplementary file 6 [file Image5.JPEG]

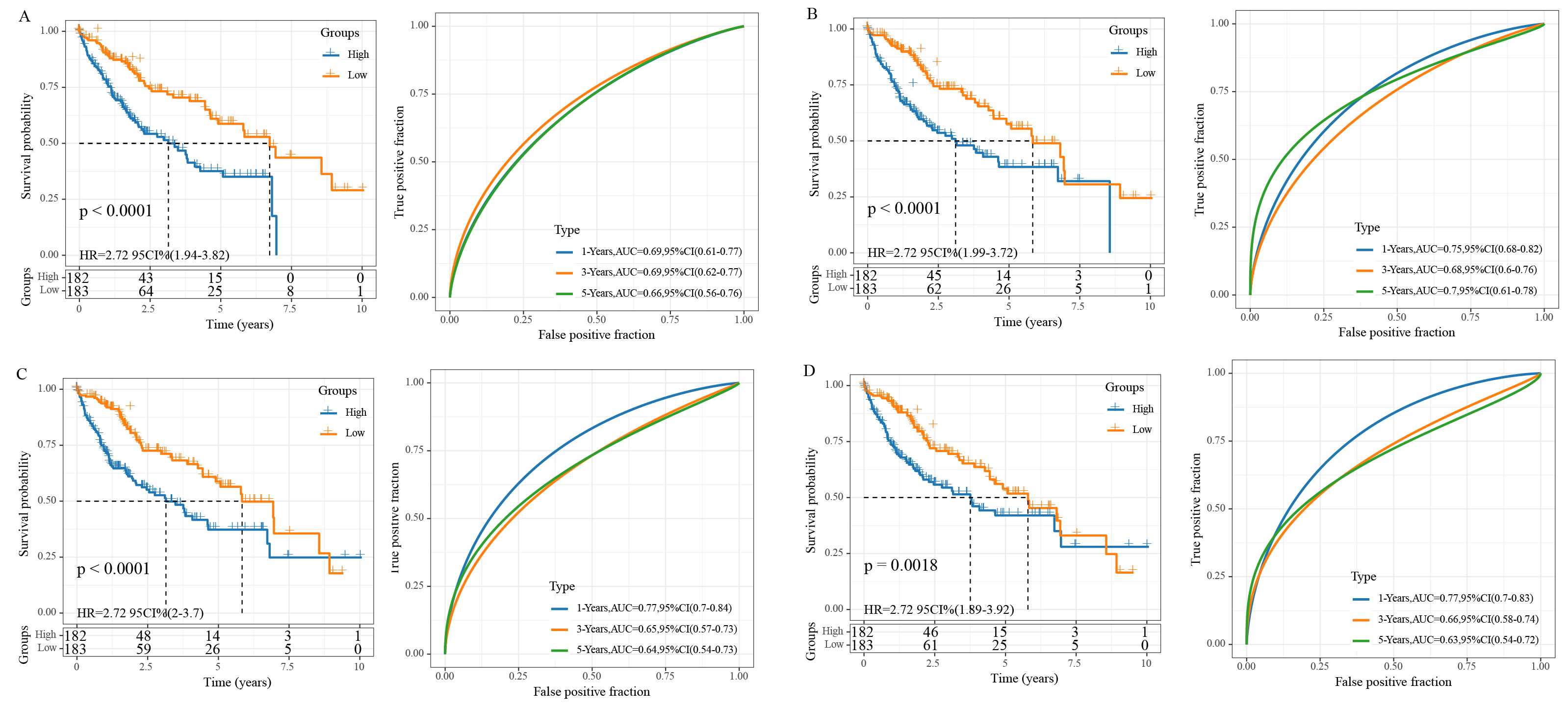

Supplement: Supplementary file 8 [file Image6.JPEG]
