# Supplementary material for: Construction of HBV gene-related prognostic and diagnostic models for hepatocellular carcinoma
Source: Front Genet. 2023 Jan 4;13:1065644. doi: 10.3389/fgene.2022.1065644 (PMC9845411; doi:10.3389/fgene.2022.1065644)
Supplement: Supplementary file 2 [file Table1.DOCX]

Table S1. Comparison of clinical features between test and train groups.

|  | Test (N=183) | Train (N=182) | P-value |
| --- | --- | --- | --- |
| **OS** |  |  |  |
| Alive | 122 (66.7%) | 113 (62.1%) | 0.421 |
| Dead | 61 (33.3%) | 69 (37.9%) |  |
| **Gender** |  |  |  |
| Female | 66 (36.1%) | 53 (29.1%) | 0.192 |
| Male | 117 (63.9%) | 129 (70.9%) |  |
| **Age** |  |  |  |
| <=60 | 79 (43.2%) | 94 (51.6%) | 0.129 |
| >60 | 104 (56.8%) | 88 (48.4%) |  |
| **T Stage** |  |  |  |
| T1 | 94 (51.4%) | 86 (47.3%) | 0.187 |
| T2 | 38 (20.8%) | 53 (29.1%) |  |
| T3 | 40 (21.9%) | 38 (20.9%) |  |
| T4 | 9 (4.9%) | 4 (2.2%) |  |
| Missing | 2 (1.1%) | 1 (0.5%) |  |
| **N Stage** |  |  |  |
| N0 | 124 (67.8%) | 124 (68.1%) | 0.116 |
| NX | 59 (32.2%) | 53 (29.1%) |  |
| N1 | 0 (0%) | 4 (2.2%) |  |
| Missing | 0 (0%) | 1 (0.5%) |  |
| **M Stage** |  |  |  |
| M0 | 127 (69.4%) | 136 (74.7%) | 0.483 |
| M1 | 2 (1.1%) | 1 (0.5%) |  |
| MX | 54 (29.5%) | 45 (24.7%) |  |
| **Stage** |  |  |  |
| I | 89 (48.6%) | 81 (44.5%) | 0.566 |
| II | 36 (19.7%) | 48 (26.4%) |  |
| III | 41 (22.4%) | 42 (23.1%) |  |
| IV | 2 (1.1%) | 2 (1.1%) |  |
| Missing | 15 (8.2%) | 9 (4.9%) |  |
| **Grade** |  |  |  |
| G1 | 29 (15.8%) | 26 (14.3%) | 0.559 |
| G2 | 93 (50.8%) | 82 (45.1%) |  |
| G3 | 53 (29.0%) | 65 (35.7%) |  |
| G4 | 6 (3.3%) | 6 (3.3%) |  |
| Missing | 2 (1.1%) | 3 (1.6%) |  |
| **Virus** |  |  |  |
| Hepatitis B | 10 (5.5%) | 12 (6.6%) | 0.885 |
| No | 79 (43.2%) | 80 (44.0%) |  |
| Missing | 94 (51.4%) | 90 (49.5%) |  |
