# Supplementary material for: Construction of HBV gene-related prognostic and diagnostic models for hepatocellular carcinoma
Source: Front Genet. 2023 Jan 4;13:1065644. doi: 10.3389/fgene.2022.1065644 (PMC9845411; doi:10.3389/fgene.2022.1065644)
Supplement: Supplementary file 7 [file Table2.DOCX]

Supplementary materials

FigS1: Enrichment analysis results of Cluster 1 network genes. A: The first 10 entries of GO-BP enrichment analysis; B: The first 10 entries of GO-CC enrichment analysis; C: The first 10 entries of GO-MF enrichment analysis; D: KEGG enrichment analysis results.

FigS2: Enrichment analysis results of Cluster 2 network genes. A: The first 10 entries of GO-BP enrichment analysis; B: The first 10 entries of GO-CC enrichment analysis; C: The first 10 entries of GO-MF enrichment analysis.

FigS3: Enrichment analysis results of Cluster 3 network genes. A: The first 10 entries of GO-BP enrichment analysis; B: The first 10 entries of GO-CC enrichment analysis; C: The first 10 entries of GO-MF enrichment analysis.

FigS4: Enrichment analysis results of Cluster 10 network genes. A: The first 10 entries of GO-BP enrichment analysis; B: The first 10 entries of GO-CC enrichment analysis; C: The first 10 entries of GO-MF enrichment analysis; D: KEGG enrichment analysis results.

FigS5: Enrichment analysis results of Cluster 11 network genes. A: The first 10 entries of GO-BP enrichment analysis; B: The first 10 entries of GO-CC enrichment analysis; C: The first 10 entries of GO-MF enrichment analysis; D: KEGG enrichment analysis results.

FigS6: Kaplan-Meier survival curves and ROC curves of previously reported prognostic models from Zheng et al. (A), Hu et al. (B), Ke et al. (C), and Liu et al. (D).

Table S1. Comparison of clinical features between test and train groups.
